# Prospective Follow-Up Assessment of Wrist Function After the Transradial Approach for Diagnostic Cerebral Catheter Angiography

**DOI:** 10.3390/jcm15062190

**Published:** 2026-03-13

**Authors:** Michael Braun, Julian Kifmann, Johannes Steinhart, Nico Sollmann, Christopher Kloth, Maria Pedro, Michal Hlavac, Jens Dreyhaupt, Meinrad Beer, Bernd Schmitz, Johannes Rosskopf

**Affiliations:** 1 Section of Neuroradiology, Bezirkskrankenhaus Guenzburg, 89312 Guenzburg, Germany; 2 Department of Diagnostic and Interventional Radiology, University Hospital Ulm, 89081 Ulm, Germany; 3 Department of Oral and Plastic Maxillofacial Surgery, Armed Forces Hospital Ulm, 89081 Ulm, Germany; 4 Department of Nuclear Medicine, University Hospital Ulm, 89081 Ulm, Germany; 5 Department of Diagnostic and Interventional Neuroradiology, School of Medicine and Health, TUM Klinikum Rechts der Isar, Technical University of Munich, 81675 Munich, Germany; 6 TUM-Neuroimaging Center, TUM Klinikum Rechts der Isar, Technical University of Munich, 81675 Munich, Germany; 7 Section of Peripheral Nerve Unit, Bezirkskrankenhaus Guenzburg, 89312 Guenzburg, Germany; 8 Department of Neurosurgery, Bezirkskrankenhaus Guenzburg, 89312 Guenzburg, Germany; 9 Institute of Epidemiology and Medical Biometry, University of Ulm, 89075 Ulm, Germany

**Keywords:** cerebral diagnostic catheter angiography, transradial access, neuroradiology, wrist function, PRWE

## Abstract

**Background:** Structural and functional alterations resulting from radial access for cerebral diagnostic catheter angiography might contribute to impaired wrist function. This study aimed to evaluate the impact of the transradial approach for diagnostic cerebral procedures on wrist function, using prospective follow-up assessments. **Methods:** Wrist function was prospectively assessed by using the Patient-Rated Wrist Evaluation (PRWE) questionnaire at baseline (pre-procedural assessment), as well as at 1-month and 3-month follow-up assessments (PRWE: 0 to 100, with 0 indicating no functional impairment). Association analyses with demographic and clinical parameters were performed using univariate logistic regression models. **Results:** During the 12-month observation period, 35 patients were enrolled in the study. At baseline, 25 patients (71.4%) reported no wrist impairment, while 10 patients (28.6%) had PRWE scores of up to 51. At the 1-month assessment, seven participants (20%) experienced a worsening in wrist function, reflected by increased PRWE scores. Of these, five patients showed deterioration exceeding the minimum clinically important difference. Another eight participants (22.9%) showed an improvement. A worsening of wrist function between the baseline and 1-month follow-up was not significantly associated with age, sex, prior neurosurgical status, body mass index (BMI), total procedure duration, dose area product, or fluoroscopy time (*p* > 0.05). At the 3-month follow-up, none of the patients reported any wrist-related impairments. **Conclusions:** In this exploratory cohort, the use of the transradial approach for cerebral angiography resulted in no wrist-related impairment at the 3-month follow-up. Transient worsening occurred in 20%, including clinically relevant cases, underscoring the need for larger studies with objective outcome measures.

## 1. Introduction

For more than a decade, the radial artery approach has been recommended as the first-choice arterial access in interventional cardiology, which is supported by several large randomized multicenter trials, with consistent evidence for improved safety and patient comfort compared to the transfemoral access [1,2]. Recently, the radial access has also been increasingly adopted for diagnostic cerebral catheter angiography, driven by its additional benefit of immediate postprocedural mobilization [3,4,5,6,7,8], which supports the shift from inpatient to outpatient care that contributes to improved cost efficiency [6,9]. The initial learning curve for neurointerventionalists has been described as steep, typically requiring 25–50 transradial accesses for cerebral angiograms, with performance benefitting from advancements in materials and anticoagulation [10]. Consequently, the implementation of patient-specific risk stratification emerges as a key area for further advancement of the transradial approach.

Wrist deterioration is a recognized risk associated with transradial access [11,12,13,14,15]. However, vascular access site complications, such as perforation, hematoma, spasms, and, most commonly, radial artery occlusion, do not appear to fully account for all observed sensory and motor complaints [16,17,18,19,20]. In addition to acute ischemic complications, chronic intimal thickening of the radial artery may be accompanied by endothelial dysfunction and a reduction in arterial diameter, raising concerns about possible adverse effects on wrist function [21,22].

Currently, upper limb dysfunction following transradial access has been addressed in only a limited number of cardiology studies [11,12,13]. Wrist deterioration is believed to be underreported, potentially due to limited detection during follow-up examinations or a general hesitancy among neurointerventionalists to properly acknowledge them [11,23]. This is particularly notable, given that wrist function plays a crucial role in many activities of daily living [24,25].

This prospective study aimed to investigate the effects of the transradial approach for diagnostic cerebral procedures on wrist function during follow-up assessments. As an objective metric, the Patient-Rated Wrist Evaluation (PRWE), a widely used and validated self-report questionnaire, was employed to assess wrist function at the baseline (pre-procedural assessments), as well as at 1-month and 3-month follow-up examinations [24,25].

## 2. Materials and Methods

### 2.1. Study Design and Patient Selection

The transradial approach serves as the primary access route for diagnostic cerebral catheter angiography at our institution. Patients who underwent transradial angiography between March 2022 and February 2023 were eligible for inclusion. All eligible patients were contacted by letter before the procedure, and all participated in the study. No patients were lost to follow-up. Exclusion criteria were lack of written informed consent or no response. Follow-up telephone interviews were conducted at 4 and 12 weeks post-procedure. In addition, the performing neuroradiologist completed a standardized questionnaire following each procedure. Prior ethical approval was granted by the institutional review board (reference number: #420/21).

### 2.2. Wrist Function

Wrist function was evaluated using the PRWE, a validated self-assessment tool commonly applied in clinical research [24,25,26]. Originally developed by MacDermid in 1996 and revised in 2019 [24,26], the PRWE comprises 15 items, with five targeting wrist pain and ten addressing functional limitations during everyday activities. Respondents rate each item on a scale from 0 (no symptoms) to 10 (maximum severity). The total score ranges from 0 to 100, with higher values indicating greater pain intensity and functional impairment.

### 2.3. Transradial Approach

Procedures for cerebral angiography were performed with the patient in the supine position. Radial access was facilitated by securing the forearm on an arm board. Under sterile conditions, 10 mL of local anesthetic (Prilocain Hydrochlorid 10 mg/mL; Xylonest^®^ 1%, Aspen Germany GmbH, Munich, Germany) was injected subcutaneously to reduce procedural pain and vasospasms. The radial access was obtained by palpation with a 20-gauge needle, followed by insertion of a 5-French access device (Glidesheath Slender^®^, Terumo Corp., Eschborn, Germany), using the Seldinger technique. Verapamil (2.5 mg; B. Braun Melsungen AG, Melsungen, Germany) and heparin (5000 U; B. Braun Melsungen AG, Melsungen, Germany) were administered intra-arterially over 2–3 min to prevent vasospasms and thrombosis. Radial angiography confirmed correct sheath placement. Selective catheterization was performed with a 0.035″ guidewire (Terumo Corporation, Eschborn, Germany) and a 5-French Simmons-2 catheter (Penumbra, Inc., Alameda, CA, USA), continuously flushed with heparinized saline. After the procedure, hemostasis was achieved by using a TR BAND^®^ radial compression device (Terumo Corp, Eschborn, Germany). No routine ultrasound or collateral circulation testing (e.g., Allen’s or Barbeau’s tests) was performed.

### 2.4. Statistical Analysis

All statistical analyses were performed using IBM SPSS Statistics (Version 30; IBM Corp., Armonk, NY, USA).

PRWE scores were reported as absolute numbers, percentages, medians (IQR) and maximum values. At first, the study cohort was stratified into participants with and without wrist impairment, defined as a PRWE score greater than zero or equal to zero, respectively. At the 1-month follow-up, changes in PRWE scores were assessed. A worsening was defined as a positive difference between the 1 month and baseline scores (Group A), while all other cases were summarized in Group B. This definition was chosen to capture even subtle changes in wrist function. Accordingly, worsening was not restricted to changes exceeding the minimal clinically important difference (MCID; ≥11.5 points) [27].

Descriptive statistics were used to summarize demographic and clinical parameters. Continuous variables were presented as means with corresponding standard deviations (SDs), as well as minimum, maximum, and median values. Categorical variables were reported as absolute numbers and percentages.

As the PRWE raw data and its changes showed a marked deviation from a normal distribution, univariate logistic regression models were used to explore potential associations. For this analysis, the worsening of wrist function between the baseline and the 1-month follow-up was defined as the dependent variable. Potential influencing factors included age, sex, prior neurosurgical status, body mass index (BMI), total procedure duration, dose-area product (DAP), and fluoroscopy time. Because of model convergence issues, the DAP was categorized into two groups, based on its median value. A *p*-value < 0.05 was considered statistically significant. Due to the explorative nature of this study, no adjustment for multiple testing was done.

## 3. Results

### 3.1. Study Sample

Over the 12-month observation period, a total of 35 patients were enrolled in this prospective study. The mean age of the cohort was 59.1 ± 10.1 years, with an age range spanning from 34 to 81 years at the time of cerebral angiography. The majority of participants were female (n = 22, 63%). In all cases, vascular access was obtained via the right radial artery. Of the included procedures, 30 (86%) were conducted as follow-up examinations after prior endovascular or neurosurgical treatments. After sheath placement, digital subtraction angiography (DSA) images of the access site were routinely obtained, allowing for the detection of dissections or perforations. Access-site complications were systematically recorded by the performing neurointerventionalist after the procedure, and no significant hematoma or vasospasm was detected. No patient underwent follow-up DSA during the follow-up period. Throughout the 3-month follow-up period, no major complications or serious adverse events were observed in any of the patients’ following CT or MR angiography.

### 3.2. Wrist Function at Baseline with Follow-Up Assessments

At the baseline, 25 patients (71.4%) reported no wrist impairment (PRWE = 0), while 10 patients (28.6%) had PRWE scores greater than 0, up to 51.

At the 1 month assessment, seven patients (20%) experienced a worsening in wrist function, reflected by increased PRWE scores (Group A). Of these, six reported new-onset wrist impairment, and one patient experienced worsening from a PRWE score of 5 to 8.5. Five patients had PRWE worsening at 1 month that exceeded the minimum clinically important difference (MCID) of ≥11.5 points) [27]. Conversely, another eight patients showed improvement at 1 month (22.9%). Six of them reported complete resolution of symptoms (PRWE = 0), while two improved from 15.5 to 14.5 and 21.5 to 20, respectively. Group B included these eight cases, as well as all patients whose PRWE remained unchanged at zero. Patients’ characteristics and procedural variables of the two groups A and B were summarized in Table 1.

At the 3-month follow-up, none of the patients reported any wrist-related impairments (all PRWE = 0), i.e., 10 patients improved from the baseline to a PRWE score of 0 at the 3-month follow-up.

In a subgroup analysis restricted to patients with a baseline PRWE of 0 (n = 25), 6 of these 25 patients (24%) demonstrated a PRWE > 0 at the 1-month follow-up, indicating that the observed worsening was not solely attributable to pre-existing wrist impairment. At 3 months, all 25 patients had returned to a PRWE of 0.

**Figure 1:**
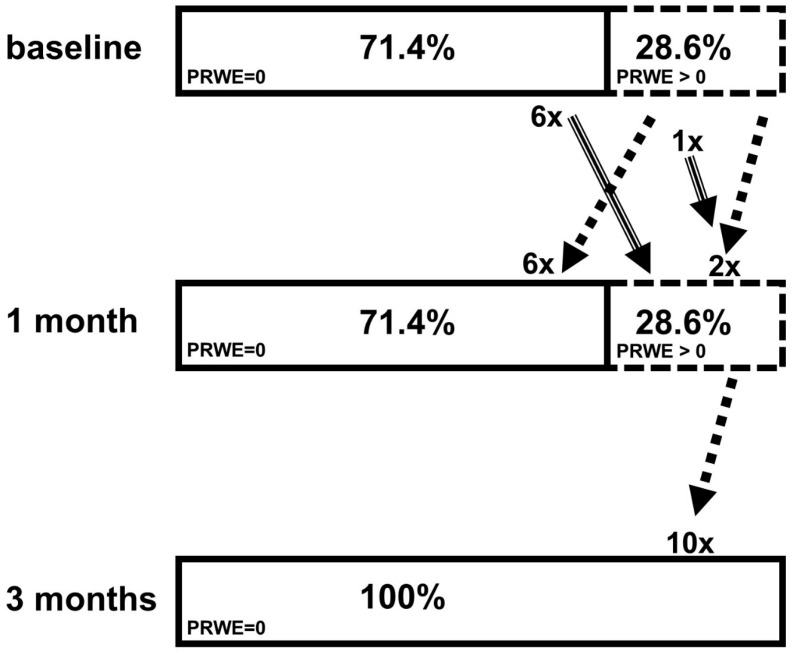
Changes in the distribution of wrist function over time, based on PRWE. The stacked bar chart shows the percentage of patients with PRWE = 0 (solid outline) and PRWE > 0 (dashed outline) at the baseline, 1-month and 3-month follow-up. Values next to the arrows indicate the absolute number. At the baseline, 71.4% of participants have no wrist dysfunction. At the 1-month follow-up, 7 patients experienced a worsening of wrist function (dashed arrow; 6 + 1) and another 8 patients showed an improvement (dotted arrow, 6 + 2). At 3 months, 100% of participants had no wrist dysfunction, i.e., 10 patients improved to a PRWE score of 0. PRWE: Patient-Rated Wrist Evaluation (range: 0–100, 0 = no impairment).

Changes in wrist function based on PRWE scores were illustrated in Figure 1 as a stacked bar chart. PRWE scores were categorized as PRWE = 0 and PRWE > 0 at the baseline, 1-month, and 3-month follow-up examinations.

At the baseline and 1- and 3-month assessments, the median (IQR) was 0.0 (0.0–5.0), 0.0 (0.0–8.5), and 0.0 (0.0–0.0), respectively.

### 3.3. Association Analyses

Univariate logistic regression models revealed no statistically significant association between a worsening of wrist function (between the baseline and 1-month follow-up) and age, sex, prior neurosurgical status, BMI, total procedure duration, dose area product, or fluoroscopy time (*p* > 0.05 each, Table 2).

## 4. Discussion

Using the PRWE, no wrist-related impairment following the transradial approach for diagnostic cerebral catheter angiography was detected at the 3-month follow-up. At the 1 month assessment 20% of patients experienced a worsening of their wrist, which was completely resolved by 3 months. This temporary impairment was not statistically significantly associated with age, sex, prior neurosurgical status, BMI, total procedure duration, dose area product, or fluoroscopy time.

The PRWE questionnaire, the Quick-DASH (a shortened version of the Disabilities of the Arm, Shoulder and Hand), and the Visual Analog Scale (VAS) are established tools for the assessment of wrist complaints [11,12,13,26,28]. The VAS is a widely used tool for quantifying subjective symptom severity, allowing patients to indicate their perceived level of discomfort on a continuous scale, typically ranging from 0 (no symptoms) to 10 (maximum severity) [28]. While the Quick-DASH evaluates function across the entire upper extremity and is frequently used in cardiology, it may lack specificity for wrist impairments. The Effects of trAnsRadial perCUtaneouS Coronary Intervention on Upper Extremity Function (ARCUS) trial reported false positive findings when using the DASH to assess upper extremity function following transradial coronary interventions [11]. In contrast, the current study employed the PRWE, a validated and wrist-specific instrument that captures both pain and functional limitations from the patient’s perspective. Its emphasis on activities of daily living may ensure clinical relevance, while its brevity and ease of use make it ideal for both routine clinical assessments and research purposes [25,26,27].

In the current study, the transradial access for diagnostic cerebral angiography did not result in wrist-related impairment at the 3-month follow-up assessment. However, an increase in PRWE was observed in 20% of participants at the 1-month follow-up. In the current study cohort, no access-site complications during sheath placement were documented. Overall, the exact mechanisms remain unclear. However, structural vascular changes, such as intimal hyperplasia and endothelial dysfunction, may play a role [21,22]. In addition, local nerve irritation or injury could also contribute to wrist dysfunction [21,22]. Moreover, as nearly one-third of participants had PRWE scores greater than zero at the baseline, this increase may partly reflect natural symptom variability. Therefore, not all observed changes can be attributed solely to access-related effects. Moreover, Zwaan et al. [11] also reported wrist dysfunction two weeks post-procedure, noting that upper extremity impairment occurred twice as frequently in the transradial group compared to the transfemoral group (n = 440 vs. 62), although no significant long-term differences were observed. In our subgroup analysis restricted to patients with a baseline PRWE of 0, 6 of these 25 patients (24%) demonstrated a PRWE > 0 at the 1-month follow-up, indicating that the observed worsening was not solely attributable to pre-existing wrist impairment. Importantly, at 3 months, all 25 patients had returned to a PRWE of 0, confirming the transient nature of these symptoms.

In comparison with the existing literature, our findings of a 20% transient worsening rate appear to fall within the expected range when upper-extremity function is systematically assessed. In the ARCUS trial [11], upper-extremity dysfunction was reported in 32.7% of patients following transradial access at 2 weeks, compared with 13.9% after transfemoral access, suggesting a higher short-term functional burden associated with radial access. In this context, the 20% transient worsening observed in our cohort lies numerically between these rates. Moreover, review data [14] on post-transradial symptoms such as hand or wrist pain report incidences of up to approximately 7.77%. However, these estimates are based on heterogeneous endpoints and are often derived from studies that did not employ dedicated functional assessment tools. This methodological variability likely contributes to lower reported rates and limits the direct comparability with studies using structured outcome measures, such as the PRWE.

The absence of wrist-related impairments at the 3-month follow-up is consistent with findings from previous studies [11,12,13]. After transradial coronary catheterization, van Leeuwen et al. [12] reported a reduction in Quick-DASH scores at one year follow-up assessments (median: 2.39 vs. 0). One possible explanation for the postprocedural improvement observed might be a response bias [29], whereby patients tend to underreport wrist-related symptoms at follow-up assessments, particularly when the overall outcome, such as successful treatment of an intracranial aneurysm, was perceived as positive. Notably, the majority of patients in our cohort (86%) underwent angiography as part of their follow-up care after prior endovascular or neurosurgical interventions. Moreover, the assessment of wrist function presents a methodological challenge [14,30,31], given that outcomes may vary depending on the timing of evaluation [12]. Early complications such as hematoma, edema, or localized discomfort following transradial access are typically transient. In the present study, all procedure-related symptoms were reported within the first four weeks, while no participant reported persistent pain or functional impairment at the 3-month follow-up evaluation. Similarly, a recent systematic review [14] found that initial sensorimotor complaints about the upper limb tended to resolve over time, further underscoring the self-limiting course of early-access site complications.

Finally, the follow-up was conducted via structured telephone interviews, rather than in-person clinical examinations, which may have additionally increased the risk of response bias. Patients may have unintentionally underreported minor or residual wrist symptoms, particularly if they perceived the primary intervention as successful overall. Consequently, it remains unclear to what extent the reported 100% resolution of symptoms at the 3-month follow-up reflects complete clinical recovery. Telephone-based assessments may be less sensitive in detecting subtle functional deficits or mild persistent symptoms that could have been identified during a standardized physical examination. Therefore, a certain degree of residual impairment cannot be definitively excluded.

Our study was not without limitations. Firstly, the relatively small sample size may have limited the statistical power to detect significant associations. As a consequence of the limited sample size and the small number of outcome events, the precision of the estimated regression coefficients is reduced, and the ability to detect small to moderate associations is limited. According to a priori sample size calculations, a sample size of 146 to 4280 patients would be required. This range depended on the predictor variables included. Secondly, as previously mentioned, response bias may have influenced the self-reported outcomes, a known challenge in studies evaluating wrist function following transradial access [12,13]. Future studies should address the potential impact of patients’ positive perception of treatment outcome as a source of response bias. Thirdly, it would have been beneficial to include additional objective parameters, such as duplex ultrasound, to assess the radial artery patency and identify access-related occlusions. However, even in the presence of radial artery occlusion, a clinically relevant perfusion deficit of the hand is generally not expected, particularly given the dual arterial supply via the ulnar artery, even when collateral circulation is less developed [19,20]. Consequently, a substantial impact on wrist-related sensorimotor symptoms would be unlikely. Nevertheless, a major limitation concerns the 20% of patients who showed worsening at the 1-month follow-up. The exact physiological mechanism underlying this functional decline could not be clearly determined, and it remains unclear whether the observed changes are primarily vascular or neurogenic in origin.

## 5. Conclusions

In this exploratory cohort, the use of transradial access for cerebral diagnostic angiography resulted in no wrist-related impairment at the 3-month follow-up. However, transient worsening at 1 month occurred in 20% (n = 7) of patients, with five exceeding the minimum clinically important difference. Although all patients returned to the baseline at follow-up, these temporary changes underline that short-term wrist impairment may occur. No significant association between worsening of wrist function and demographic or clinical parameters was identified, possibly reflecting the limited sample size. Overall, the findings indicate a favorable safety of the transradial technique. Future studies with larger cohorts and objective outcome measures may further strengthen and validate these observations.

## Figures and Tables

**Table 1:** Patients’ characteristics and procedural variables.

Variable	Group A (n = 7) ^1^	Group B (n = 28) ^2^
**Age (years) ^3^**	54.1 ± 7.9; 53 [47.0–71.0]	60.4 ± 10.4; 62.5 [34.0–81.0]
**Sex (m/f) ^4^**	3/4	10/18
**Angiography before/after ^5^** **endovascular treatment**	1/6	3/25
**BMI (kg/m^2^) ^6^**	26.2 ± 3.0; 26.4 [22.2–30.6]	28.3 ± 5.7; 27.7 [19.1–41.5]
**DAP (µGym^2^) ^7^**	3334.6 ± 2356.5; 2440.6 [1143.8 8295.6]	3560.4 ± 2951.8; 2558.9 [1146.7–13,025.3]
**Total duration (min) ^8^**	23.9 ± 8.4; 24.0 [12.0–37.0]	21.8 ± 12.5; 19.5 [5.0–52.0]
**Fluoroscopy time** **(min) ^9^**	9.0 ± 3.0; 8.6 [4.2–12.9]	8.3 ± 4.9; 7.1 [2.4–20.0]

^1^ Group A consisted of the seven patients with a deterioration in wrist function, indicated by a higher PRWE score at 1 month compared to the baseline. ^2^ Group B comprised all other cases. ^3^ Mean ± standard deviation; median [min–max]. ^4^ Male/female. ^5^ Number of patients before/after endovascular treatment. ^6^ Body mass index. ^7^ Dose area product. ^8^ Total duration of the procedure in the angiosuite. ^9^ Fluoroscopy time of the procedure in the angiosuite.

**Table 2:** Results of the association analysis, using univariate logistic regression models.

Variable	Odds Ratio ^1^	95–CI ^2^	*p*-Value ^3^
**Age (years)**	0.939	0.862–1.024	*p* = 0.154
**Sex (m/f) ^4^**	1.350	0.250–7.278	*p* = 0.727
**Angiography before/after** **endovascular treatment**	1.389	0.122–15.813	*p* = 0.791
**BMI (kg/m^2^) ^5^**	0.920	0.768–1.101	*p* = 0.362
**DAP (µGym^2^) ^6^**	0.750	0.141–3.985	*p* = 0.736
**Total duration (min) ^7^**	1.015	0.948–1.087	*p* = 0.673
**Fluoroscopy time** **(min) ^8^**	1.033	0.865–1.233	*p* = 0.722

The univariate logistic regression models showed no significant association between wrist function worsening (baseline to 1 month) and demographic and clinical variables. ^1^ Values represent odds ratio with ^2^ 95% confidence intervals (CI) and ^3^ *p*-values. ^4^ Male/female. ^5^ Body mass index. ^6^ Dose area product categorized by median, due to model convergence issue. ^7^ Total duration of the procedure in the angio-suite. ^8^ Fluoroscopy time of the procedure in the angiosuite.

## Data Availability

The data that support the findings of this study are available from the corresponding author upon reasonable request. Due to ethical and legal restrictions related to the protection of participant privacy and confidentiality, the raw data cannot be made publicly available.

## Abbreviations

The following abbreviations are used in this manuscript:

DAP	Dose Area Product
DASH	Disabilities of the Arm, Shoulder, and Hand
PRWE	Patient-Rated Wrist Evaluation
Quick-DASH	Shortened version of Disabilities of the Arm, Shoulder, and Hand
VAS	Visual Analog Scale
